# Dislocation of ulnar side all four carpometacarpal joints – a case report

**DOI:** 10.1080/23320885.2021.1910039

**Published:** 2021-04-10

**Authors:** Ram K. Shah, Abhisek Ranjan, Niraj K. Shah

**Affiliations:** Department of Trauma & Orthopaedic Surgery, Janakpur Trauma Hospital, Janakpurdham, Nepal

**Keywords:** Dislocation, injury rehabilitation, hand

## Abstract

Carpometacarpal joint dislocations of the hand are exceptional; their diagnosis is sometimes difficult and may go unnoticed especially in a patient with polytrauma. The functional prognosis depends on the precocity of diagnosis and the quality of the reduction and rehabilitation. A successful result has been reported.

## Background

Consecutive ulnar side four carpometacarpal (CMC) joint dislocations of the hand are very rare. These injuries mainly occur in young adults and represent less than 1% of all injuries of the hand. Simultaneous CMC dislocations may be dorsal or volar. Dorsal dislocations are more frequent. The reason behind dorsal dislocations being more common is that the stronger static (dorsal ligaments) and dynamic (wrist extensors) restraints cause the failure of bone dorsally, with frequent rupture of the volar ligaments [1,2]. Increased mobility on the ulnar side may predispose to a higher frequency of injury. The stability at the finger CMC joint is provided by a system of four ligaments, namely the dorsal metacarpal, the palmar metacarpal and the two sets of interosseous ligaments [3]. CMC joint dislocations can be treated by closed reduction and immobilizations, closed reduction, internal fixation or open reduction and internal fixation [4]. However, in the case of closed reduction, there is a higher risk of redislocations of the CMC joints, as compared to open reduction.

Severe swelling associated with these injuries and overlapping of bones on a radiograph of the wrist and hand can cause misdiagnosis of CMC dislocations. The purpose of this clinical case report is to highlight this unusual injury to avoid missing diagnosis.

## Case presentation

A 47-year-old male person was admitted to the emergency department of this hospital with complaints of severe pain, discomfort and inability to move his dominant right hand and fingers following a motorbike accident. An initial clinical examination found that he was hemodynamically stable.

**Figure 1.**
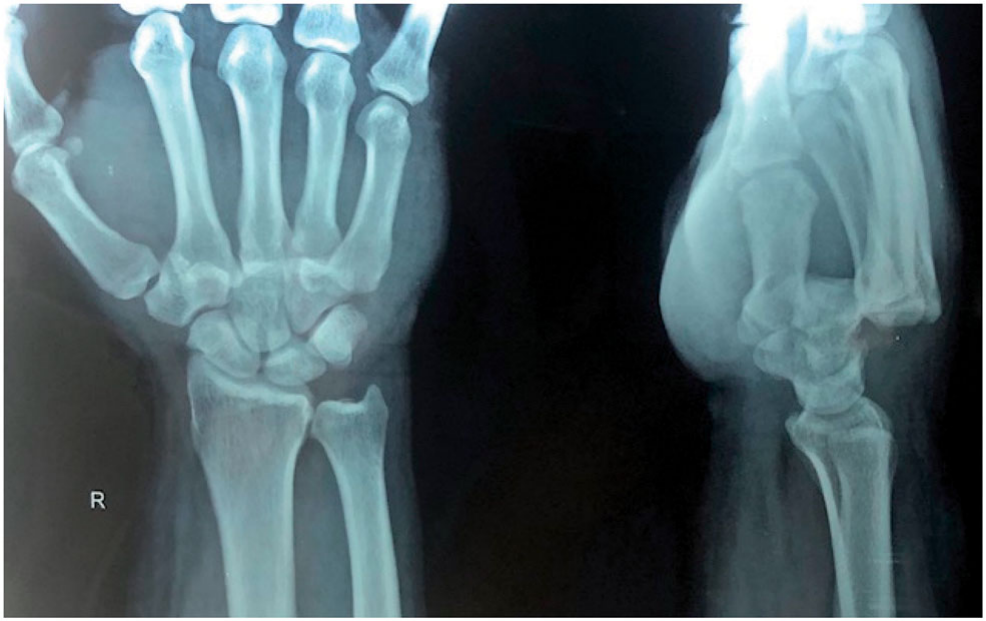
PA and lateral views of wrist.

An examination of his right upper limb showed significant edema and a clear deformity on the dorsal side of his right hand with no signs of nerve compression. The vascular examination was normal. The radiographs of his wrist showed dorsally dislocated ulnar side four digits at the level of the CMC joint (Figure 1).

**Figure 2.**
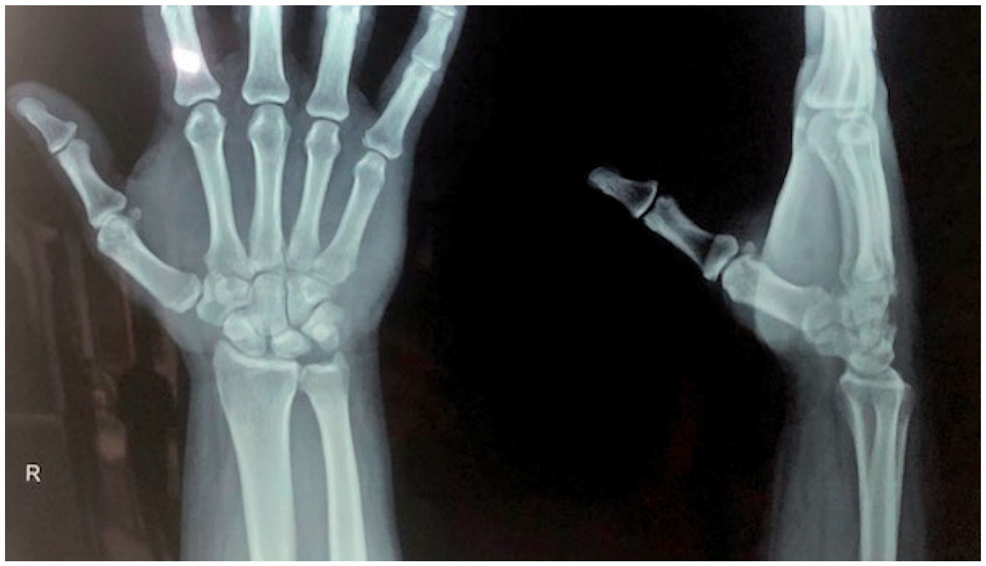
Follow up X-rays of wrist PA & lateral views CMC is in alignment.

The following day, he was taken to the operation theater and prepared for open reduction and internal fixation. But, the initial attempt of closed reduction with traction and counter traction was unexpectedly successful and achieved stable reduction which was confirmed with ‘C’-arm Xray. It had concentric reduction in the anteroposterior, lateral and oblique views. The stability of the reduction was assessed with a full range of movement at the wrist joint, CMC joint and Metacarpo-phalangeal joints, which confirmed that it was quite stable (Figure 2). Subsequently, a plaster cast was applied dorsally for 2 weeks and the reduction was reassessed in two consecutive weeks in the fracture clinic with X-ray assessment.

**Figure 3.**
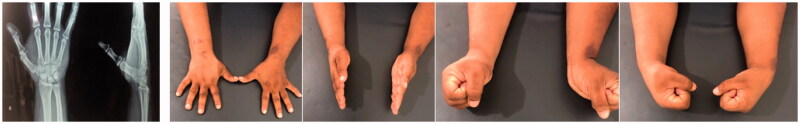
Follow up radiographs of wrist and clinical photos after 2 months.

After 2 weeks, it was changed into a thermoplastic cockup splint by a hand surgeon, followed by formal hand physiotherapy after 6 weeks. Functional assessment following reduction was assessed at 6 weeks using the DASH (Disability of Arm, Shoulder and Hand) questionnaire. He achieved grip strength and wrist mobility up to near normal over two months (Figure 3).

## Discussion

Dislocations of all four ulnar CMC joints are rare. These injuries mainly occur in young adults. Road traffic accidents and violent trauma are the main etiology [4]. The clinical diagnosis is sometimes difficult due to edema that takes place early and masks the deformity. This injury may be missed in an acute setting in a busy accident and emergency unit. Swelling around the wrist with shortening of the knuckle should alert clinicians to the possibility of such an injury [1,2,5]. In these cases, radiology remains an important diagnostic benefit, although the interpretation of images is sometimes difficult. On routine AP view, evaluation of CMC joints is done by parallel ‘M lines’ as described by Glula; overlap of joint surfaces, loss of parallelism and asymmetry at the CMC joints should raise the suspicion of the possibility of a subtle CMC injury. This article highlights the importance of a high index of suspicion, a true lateral radiograph and careful evaluation of radiographs in diagnosing these injuries.

A CT scan can also be recommended for better analysis of the lesions and to detect any associated lesions unnoticed by standard radiographs.

In order to evaluate the results following intervention in carpometacarpal injuries, several outcome measures have been used, including patient-based, subjective measures and radiographic assessments. Patient-based subjective measures include the 36-Item Short Form Health Survey (SF-36), DASH Questionnaire, Patient-rated Wrist Hand Evaluation (PRWE), Michigan Hand Outcome Questionnaire and the Jebsen–Taylor hand function test (JTT). The optimal tool should however be easily administered and comprehensive in terms of function, pain, disability and response to surgery. Since a gold standard outcome tool is not currently identified, a combination of currently used subjective outcome measures along with radiographic parameters needs to be relied upon [6].

## Conclusion

Dislocations of the carpometacarpal joint of the hand are exceptional; their diagnosis is sometimes difficult and may go unnoticed, especially in a patient with polytrauma. The functional prognosis depends on the precocity of diagnosis, quality of reduction and rehabilitation.
